# Authentication of *Aspergillus parasiticus* strains in the genome database of the National Center for Biotechnology Information

**DOI:** 10.1186/s13104-021-05527-6

**Published:** 2021-03-23

**Authors:** Perng-Kuang Chang

**Affiliations:** Southern Regional Research Center, Agricultural Research Service, U. S. Department of Agriculture, 1100 Robert E. Lee Boulevard, New Orleans, LA 70124 USA

**Keywords:** *Aspergillus parasiticus*, *Aspergillus flavus*, Species identification, Genome, Single nucleotide polymorphism, SU-1, NRRL2999, NRRL3357

## Abstract

**Objective:**

The use of genome sequences from strains authenticated to correct species level is a prerequisite for confidently exploring the evolutionary relationship among related species. *Aspergillus* strains erroneously curated as *Aspergillus oryzae* and *Aspergillus fumigatus* have been noticed in the National Center for Biotechnology Information (NCBI) genome database. *Aspergillus parasiticus* is one of several aspergilli that produce aflatoxin, the most potent carcinogenic mycotoxin known up to now. To ensure that valid conclusions are drawn by researchers from their genomics-related studies, molecular analyses were carried out to authenticate identities of *A. parasiticus* strains in the NCBI genome database.

**Results:**

Two of the nine supposedly *A. parasiticus* strains, E1365 and NRRL2999, were found to be misidentified. They turned out to be *Aspergillus flavus* based on genome-wide single nucleotide polymorphisms (SNPs) and genetic features associated with production of aflatoxin and cyclopiazonic acid. NRRL2999 lacked the additional partial aflatoxin gene cluster known to be present in its equivalent strain, designated as SU-1, and shared a very low total SNPs count specifically with *A. flavus* NRRL3357 but not with other *A. flavus* isolates. Therefore, the mislabeled NRRL2999 strain actually is a clonal strain of *A. flavus* NRRL3357, whose genome was first sequenced in 2005.

**Supplementary Information:**

The online version contains supplementary material available at 10.1186/s13104-021-05527-6.

## Introduction

*Aspergillus parasiticus* is a saprophytic fungus found in soil and decayed plant materials. It was first isolated, from dead mealy bugs in Hawaiian sugarcane, and characterized by Speare in 1912 [1]. *A. parasiticus* was originally classified as a subspecies of *Aspergillus flavus*, because of its morphological resemblance to *A. flavus*. Nonetheless, *A. parasiticus* can be distinguished from *A. flavus* based on other characteristics, such as darker green conidial heads and more rough conidium surface ornamentation [2].

Aflatoxin B_1_ is the most carcinogenic mycotoxin known [3]. Nearly all *A. parasiticus* isolates are highly aflatoxigenic but abilities of *A. flavus* isolates to produce aflatoxin vary greatly. Evidence of the aflatoxin biosynthesis gene cluster, as well as its complete characterization, were revealed in *A. parasiticus* [4]. Every known contributing enzyme to date and the majority of biosynthesis genes were first characterized in *A. parasiticus* [5]. Since the early 2000s, *A. flavus* has gradually replaced *A. parasiticus* in studies dealing with population and genetic diversity, pathogen-host interactions, and biological control; however, *A. parasiticus* remains an important research subject because of its potential to co-infect crops such as corn and peanuts.

Rapid advancements in massive parallel sequencing in the genomic era have accelerated resolution of genome information. Genome sequences of over 100 *A. flavus* isolates and nine *A. parasiticus* strains have been made publicly available from NCBI (https://www.ncbi.nlm.nih.gov/genome). These genome assemblies, along with those from closely related aspergilli such as *A. oryzae* and *A. sojae*, are invaluable resources for comparative genomics studies involving the investigation of chromosomal structure, evolutionary relationship, and genetic variations in relation to aflatoxin-producing capabilities of aspergilli [6–8].

In the course of analyzing genome sequences deposited in the NCBI genome database, discrepancies were revealed for identities of some of the strains designated as *A. parasiticus*, necessitating a closer examination to (1) uncover the misidentified strains, (2) reveal their correct species identities, and (3) notify NCBI so that researchers would not risk erroneous conclusions from genomics studies involving these misidentified strains.

## Main text

### Materials and methods

#### Determination of genome-wide single nucleotide polymorphisms

Genome sequences of *A. parasiticus* and *A. flavus* were retrieved from the NCBI genome database (https://www.ncbi.nlm.nih.gov/genome). Table 1 lists *A. parasiticus* and *A. flavus* strains along with their genome information used in the study. The program progressiveMauve of Mauve (http://darlinglab.org/mauve/mauve.html), a system for multiple genome alignments [9], was used to obtain genome-wide single nucleotide polymorphisms (SNPs) from analyzed strains, here named total SNPs. Aligning all paired genome sequences to extract total SNPs was performed using a custom JavaScript.

**Table 1:** *Aspergillus* strains and genome sequences used in this study

*A. parasiticus*^a^	Size (Mb)	WGS	*norB-cypA* region^d^	AF/CPA clusters^e^
68–5	30.14	LOAP01	Intact	Y/N
CBS117618	38.39	SWCZ01	Intact	Y/N
E1348	39.35	SJFE01	Intact	Y/N
E1365	37.77	SJFF01	II	Y/Y
E1337	39.15	SJFC01	Intact	Y/N
E1443	41.45	SJFK01	Intact	Y/N
E1319	38.94	SJFB01	Intact	Y/N
NRRL2999	37.05	CP051027.1–CP051034.1^c^	II	Y/Y
SU-1 (JCVI)	39.47	JZEE01	Intact	Y/N
SU-1 (MSU)	40.06	JMUG01	Intact	Y/N
*A. flavus*^b^				
AF12	38.03	NLCN01	I	Y/Y
AF70	38.05	NLCM01	I	Y/Y
AZS04M2A	38.27	NLCL01	I	Y/Y
CA14	37.70	QQZZ01	II	Y/Y
CS0504	36.98	NLCK01	II	Y/Y
CS0540	36.94	NLCJ01	II	Y/Y
CS1137	37.42	NLCI01	II	Y/Y
NRRL3357	36.89	AAIH02	II	Y/Y
NRRL3357	37.75	CP044616.1–CP044623.1^c^	II	Y/Y
WRRL1519	38.04	NPKL01	II	Y/Y

^a^ Designation “E” indicates its Ethiopian origin. SU-1 was independently sequenced by John Linz’s group at Michigan State University (MSU) and a collaborative group of researchers from USDA-ARS and J. Craig Venter Institute (JCVI)

^b^AF12, AF70, and AZS04M2A are S-morphotype *A. flavus* isolates that produce small sclerotia. Other isolates are L-morphotype *A. flavus*

^c^Designations represent eight (I to VIII) chromosomes

^d^I and II indicate type I and type II deletions, which are independent events, in the *norB-cypA* region of the aflatoxin gene cluster [10, 11]. Type I deletion is found in aflatoxigenic S-morphotype *A. flavus* while type II deletion is found in aflatoxigenic L-morphotype *A. flavus*. S- and L-morphotype strains produce large and small sclerotia, respectively

^*e*^*AF* aflatoxin, *CPA* cyclopiazonic acid. Presence of complete respective gene clusters: *Y* yes and *N* no

#### Differentiation of *A. parasiticus* and *A. flavus* based on unique genetic features

Analyses of genetic features of genome sequences were performed via CoGe (Comparative Genomics, https://genomevolution.org/coge/), an online platform for retrieval and comparison of genomic information. For the determination of the *norB-cypA* deletion patterns in the aflatoxin gene cluster [10, 11], sequences corresponding to that region from *A. parasiticus* SU-1, and *A. flavus* NRRL3357 (L-morphotype) and AF12 (S-morphotype) were used as alignment templates for *Aspergillus* genome sequences. For the determination of aflatoxin and cyclopiazonic acid (CPA) gene clusters, respective accessioned gene clusters from *A. flavus* AF36 (GenBank Accession numbers: AY510455 and JN712209) were used in sequence alignment.

### Results and discussion

#### Discrepancies in total SNPs counts among *A. parasiticus* strains

**Fig. 1:**
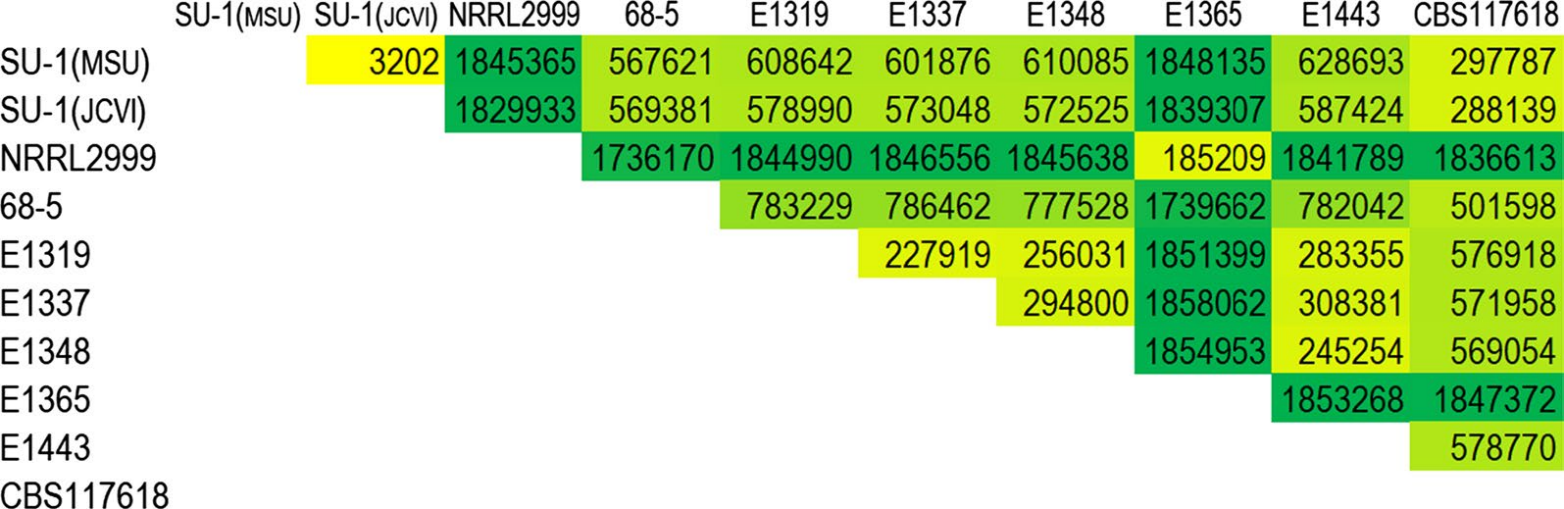
Total SNPs from paired genome sequence comparisons among *A. parasiticus* strains

**Fig. 2:**
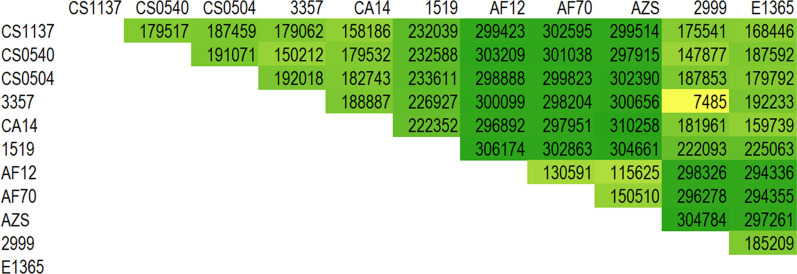
Total SNPs from paired genome sequence comparisons among NRRL2999, E1365, and other *A. flavus* S- and L-morphotype isolates

Ten *A. parasiticus* genome sequences, which supposedly were derived from eight independent isolates, are available from NCBI (Table 1). These sequences include five from Ethiopian peanut isolates [12], one from a Georgia, USA peanut isolate 68–5 [13], one from an isolate (CBS117618) collected from the leaf of an Argentinian wild peanut species (*Arachis correntina*) used for an *Aspergillus* whole-genus sequencing project [14], and three from the same isolate (SU-1 = NRRL2999) that had been independently sequenced by three groups because of the aforementioned significance in the aflatoxin biosynthesis research [15, 16]. Resolved genome sizes of these *A. parasiticus* strains range from 30.0 to 41.5 Mb, and most fall within a range of 38.0–40.0 Mb. The extraordinarily small size of strain 68–5 (> 20% less than others) likely resulted from poor library construction and/or an inadequate sequencing read coverage. Total SNPs among Ethiopian isolates, with the exception of E1365, ranged from 227,929 to 303,881 (Fig. 1). In contrast, total SNPs from E1365 compared with others were nearly 6- to eightfold higher, ranging from 1,851,399 to 1,858,062, which suggests that E1365 is not an *A. parasiticus* strain. The SU-1 genome, independently sequenced by two research groups and here designated as MSU and JCVI, had a total SNPs count of 3,202 (Fig. 1). This low number of nucleotide variations likely arises from a combination of sequencing errors, mutations accumulated over time, and subculturing. The total SNPs count from SU-1 compared with CBS117618 was comparable to those observed among the four Ethiopian isolates. *A. parasiticus* SU-1/NRRL2999 was originally isolated from a Ugandan peanut in 1961 [17]. Over the past decades, this isolate has been given other strain designations, such as Austwick strain V. 3734/10, Hodges M-3, SYS-4, ATCC56775, ATCC26692, CMI91019b, NRRL5862, and SRRC143, depending on whether it was a transfer to another laboratory or was deposited into a culture collection center [18, 19]. Despite SU-1/NRRL2999 supposedly being the same strain, total SNPs from their comparisons, however, were 1,829,933 and 1,845,365. Total SNPs count is a good indicator for assigning isolates to species level. For example, total SNPs between *A. flavus* L-morphotype and S-morphotype isolates are about 300,000 while counts from the same morphotype isolates are much lower (20, also Fig. 2). Additionally, total SNPs for three *A. nidulans* strains were around 34,000 (data not shown). For 18 *A. fumigants* strains, there was a wide range of total SNPs observed although less than 170,000 (Additional file 1: Fig. S1). Therefore, these extraordinarily high counts of total SNPs, which represented approximately only 95.3% genome sequence identity, indicate that the deposited NRRL2999 is a completely different species from SU-1.

#### Lack of typical *A. parasiticus* genetic features in strains E1365 and NRRL2999

*A. parasiticus* generally can be differentiated from *A. flavus* based on macro- and micro-morphological characteristics. Moreover, *A. parasiticus* is unlike *A. flavus* in that it produces aflatoxins G_1_ and G_2_, in addition to B_1_ and B_2_, but it does not produce CPA. Molecular events underlying these differences in mycotoxin production have been well characterized. In *A. flavus*, the CPA biosynthesis gene cluster resides next to the aflatoxin biosynthesis gene cluster in a subtelomeric region on chromosome III, while in *A. parasiticus* the CPA gene cluster is mostly deleted [21]. Additionally, two early pathway genes required for formation of G_1_ and G_2_, *norB* and *cypA*, are intact in *A. parasiticus*, but in *A. flavus* S- and L-morphotype strains, there are deletions in these genomic regions that render the strains incapable of G-type aflatoxin production [22, 23]. A schematic representation of the *norB-cypA* region in the aflatoxin gene clusters of *A. parasiticus* and *A. flavus* S- and L-morphotype isolates are shown (Additional file 1: Fig. S2). Both E1365 and NRRL2999 have the unique deletion belonging to L-morphotype strains; that is, the type II *norB-cypA* deletion (Table 1). Additionally, a complete 17-kb CPA gene cluster was located on contig SJFF01000023.1 of strain E1365 from nucleotides 1,918,903 to 1,935,762 and on Chromosome III of NRRL2999 (CP051029.1) from nucleotides 5,182,875 to 5,199,734, respectively. In contrast, no contigs of large portions homologous to the CPA gene cluster were found in either of the SU-1 genome sequences (Additional file 1: Fig. S3).

#### NRRL2999 lacks the partial duplicate aflatoxin gene cluster present in SU-1 and shares low total SNPs count with *A. flavus* NRRL3357

Another line of evidence arguing against NRRL2999 being an *A. parasiticus* strain came from its missing the SU-1 partial duplicate aflatoxin gene cluster that contains homologs of *aflR-aflJ-adhA-estA-norA-ver1* and *omtB* [24]. A near-duplicate copy of *ver1* in *A. parasiticus* NRRL2999 (= SYS-4) also has been reported [25, 26]. Sequence alignment showed that in addition to the complete aflatoxin gene cluster, a large (14.6 kb) portion of the 17.4-kb genomic fragment that contains the partial aflatoxin gene cluster (GenBank Accession number: AF452809) was located on each of the two SU-1(JCVI) contigs, JZEE01000205.1 from nucleotides 1 to 11,696 and JZEE01000720.1 from nucleotides 5 to 2915, respectively. Similarly, various contigs of SU-1(MSU), the sizes of which were in total of 16.7 kb, were found beside its complete aflatoxin gene cluster. However, no sequence homologous to the partial aflatoxin gene cluster was present in the NRRL2999 genome sequence, which was assembled at the chromosome level, except for a complete aflatoxin gene cluster on chromosome III. NRRL2999 like E1365, shared similar total SNPs counts with other *A. flavus* S- and L-morphotype isolates except NRRL3357 (Fig. 2). This shared low total SNPs count indicates that the misidentified NRRL2999 strain is indeed a clone of *A. flavus* NRRL3357 [27].

## Limitations

Ugandan and Argentinian isolates of *A. parasiticus*, SU-1 and CBS117618, shared a total SNPs count of approximately 290,000 well within reasonable range for *A. flavus*. However, the four Ethiopian isolates and one of the American (68–5, taking into consideration of its 20% less than average genome size) isolates in comparison had total SNPs twice that count (approximately 580,000). Strikingly, the Ethiopian and American isolates shared total SNPs counts three times that of the Ugandan and Argentinian isolates. Whether the observed evolutionary distance in terms of total SNPs resulted from geographic separation and niche adaptation, or the Ethiopian and American isolates are not *A. parasiticus* but very closely-related aspergilli, is not clear. With regard to NRRL2999 and the genome sequence associated with it, the source of error, whether it was due to an erroneously provided stock culture, or a mix-up of sequencing samples of *Aspergillus* isolates, or a mislabeling of sequence read datasets, cannot be pinpointed.

## Supplementary Information

**Additional file 1: Fig. S1**. Total SNPs from paired genome sequence comparisons among 18 *A. fumigatus* isolates. **Fig. S2**. Schematic representation of the *norB-cypA* region in the aflatoxin gene cluster of *A. parasiticus* and *A. flavus*. Arrows indicate direction of gene transcription. Dashed lines indicate deleted sequences. Deletion patterns, type I and type II, correspond to *A. flavus* S- and L-morphotype isolates. **Fig. S3**. CoGeBlast of the CPA gene cluster sequence of *A. flavus* AF36 against genome sequences of E1365, NRRL2999, and SU-1.

## Data Availability

All major data generated or analyzed during this study are included in this article. Those not presented are available from the author on reasonable request. The following are links to NCBI *Aspergillus* genome databases used in this study. *Aspergillus parasiticus* genome database. https://www.ncbi.nlm.nih.gov/genome/browse/#!/eukaryotes/12976/. *Aspergillus flavus* genome database. https://www.ncbi.nlm.nih.gov/genome/browse/#!/eukaryotes/360/. *Aspergillus nidulans* genome database. https://www.ncbi.nlm.nih.gov/genome/browse/#!/eukaryotes/17/. *Aspergillus fumigatus* genome database https://www.ncbi.nlm.nih.gov/genome/browse/#!/eukaryotes/18/. The GenBank Accession number for the *norB-cypA* region in *A. parasiticus* SU-1 is AY371490.1 (https://www.ncbi.nlm.nih.gov/nuccore/AY371490.1). The GenBank Accession numbers for aflatoxin and cyclopiazonic acid gene clusters of *A. flavus* AF36 are AY510455 (https://www.ncbi.nlm.nih.gov/nuccore/AY510455) and JN712209 (https://www.ncbi.nlm.nih.gov/nuccore/JN712209), respectively.

## Abbreviations

CPA: Cyclopiazonic acid
NCBI: The National Center for Biotechnology Information
SNP: Single nucleotide polymorphism
JCVI: J. Craig Venter Institute
MSU: Michigan State University
ATCC: American Type Culture Collection
NRRL: ARS (Agricultural Research Service) Culture Collection
SRRC: Southern Regional Research Center Culture Collection
CBS: Dutch Centraalbureau voor Schimmelcultures
CMI: Commonwealth Mycological Institute
