# Supplementary material for: Authentication of Aspergillus parasiticus strains in the genome database of the National Center for Biotechnology Information
Source: BMC Res Notes. 2021 Mar 23;14:111. doi: 10.1186/s13104-021-05527-6 (PMC7989025; doi:10.1186/s13104-021-05527-6)
Supplement: Supplementary file 1 — Additional file 1: Fig. S1. Total SNPs from paired genome sequence comparisons among 18 A. fumigatus isolates. Fig. S2. Schematic representation of the norB-cypA region in the aflatoxin gene cluster of A. parasiticus and A. flavus. Arrows indicate direction of gene transcription. Dashed lines indicate deleted sequences. Deletion patterns, type I and type II, correspond to A. flavus S- and L-morphotype isolates. Fig. S3. CoGeBlast of the CPA gene cluster sequence of A. flavus AF36 against genome sequences of E1365, NRRL2999, and SU-1. [file 13104_2021_5527_MOESM1_ESM.pdf]

	AAHF01.1	ABDB01.1	AFXL01.1	AFXM01.1	AZZA01.1	BCGY01.1	JAAAPZ01.1	JAAAQA01.1	JAAAQB01.1	JAAAQC01.1	JAAAQD01.1	JHOI01.1	LWRT01.1	LWRU01.1	MCQI02.1	NKHS01.1	NKHT01.1	VBRB01.1
**AAHF01.1**		53004	59481	69961	74039	48462	60344	77940	87394	48159	58535	144700	60585	58568	139401	67761	90103	58965
**ABDB01.1**			17350	56088	59085	42712	44152	65920	77969	31136	18684	143270	43319	40617	135585	48787	76591	54552
**AFXL01.1**				72617	74509	50790	43183	68990	74063	32795	18164	157522	35411	37636	151769	38746	77497	62416
**AFXM01.1**					39935	59266	50544	24509	48964	60536	66812	157189	63028	67590	148802	66358	41809	69114
**AZZA01.1**						61476	54991	43220	36528	59614	66859	160526	68758	66495	147404	75807	48186	64452
**BCGY01.1**							54318	61520	70430	48609	50241	153378	52507	51719	142968	57439	67927	54326
**JAAAPZ01.1**								52036	56234	38904	42515	149550	54789	50993	144342	54478	55046	58871
**JAAAQA01.1**									53347	64425	67835	154987	68669	71979	150901	71426	36183	70041
**JAAAQB01.1**										65252	67166	164122	73517	72571	159359	74960	44292	72368
**JAAAQC01.1**											34099	143385	41443	39800	139610	42665	62109	53983
**JAAAQD01.1**												152230	39226	41067	148388	42041	65958	58454
**JHOI01.1**													155275	152055	20744	158835	162961	152985
**LWRT01.1**														27849	144283	25009	71644	57213
**LWRU01.1**															145434	17134	70538	56576
**MCQI02.1**																149200	156356	145823
**NKHS01.1**																	73674	62215
**NKHT01.1**																		70644
**VBRB01.1**																		

Fig. S1 Total SNPs from paired genome sequence comparisons among 18 *A. fumigatus* isolates.

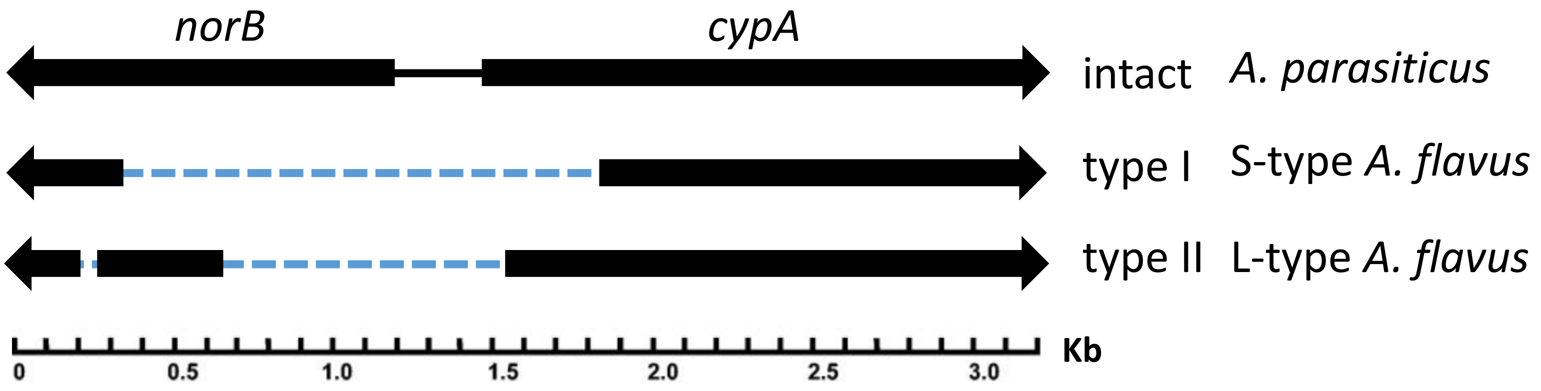

Fig. S2 Schematic representation of the *norB*-*cypA* region in the aflatoxin gene cluster of *A. parasiticus* and *A. flavus*. Arrows indicate direction of gene transcription. Dash lines indicate deleted sequences. Deletion patterns, type I and type II, correspond to *A. flavus* S- and L-morphotype isolates.

E1365

Chr	Position	HSP#	Length	E-value	Perc ID
SJFF01000023.1	1918903	1	16860	0.0	99.8%
SJFF01000021.1	20482	2	105	2e-06	76.1%

NRRL2999

Chr	Position	HSP#	Length	E-value	Perc ID
CP051029.1	5182875	1	16860	0.0	99.8%

SU-1 (JCVI)

Chr	Position	HSP#	Length	E-value	Perc ID
JZEE01000727.1	4541	1	1754	0.0	91.5%
JZEE01000168.1	5299	2	607	0.0	85.8%
JZEE01000727.1	12654	3	446	7e-149	87.6%
JZEE01000232.1	229532	4	107	1e-07	76.6%
JZEE01000251.1	155854	5	74	7e-06	79.7%
JZEE01000251.1	156496	6	47	7e-06	87.2%

SU-1 (MSU)

Chr	Position	HSP#	Length	E-value	Perc ID
JMUG01000976.1	8349	1	1754	0.0	91.5%
JMUG01002156.1	681	2	607	0.0	85.8%
JMUG01000976.1	236	3	446	8e-149	87.6%
JMUG01000015.1	29000	4	107	1e-07	76.6%
JMUG01000163.1	44361	5	47	7e-06	87.2%
JMUG01000163.1	45003	6	74	7e-06	79.7%

Fig. 3 CoGeBlast of the CPA gene cluster sequence of *A. flavus* AF36 against genome sequences of E1365, NRRL2999, and SU-1.
